# First experience of robotic spleen-preserving distal pancreatectomy in a child with insulinoma

**DOI:** 10.1186/s12957-017-1265-6

**Published:** 2017-11-09

**Authors:** Ming-Gen Hu, Yuan-Hong Xiao, Dong-Da Song, Guo-Dong Zhao, Yan-Zhe Liu, Zheng Wang, Hao-Yu Li, Rong Liu

**Affiliations:** 1 Department of Surgical Oncology (HBP), Chinese PLA General Hospital, Beijing, 100853 China; 2 Department of Pediatric Surgery, Chinese PLA General Hospital, Beijing, 100853 China

**Keywords:** Robotic surgery, Insulinoma, Distal pancreatectomy, Pediatric surgery

## Abstract

**Background:**

An insulinoma is a functional neuroendocrine pancreatic tumor, and surgical resection is indicated. Robot-assisted laparoscopic surgeries have been shown to be generally safe and feasible for treatment of pediatric cases of urologic and digestive disease.

**Case presentation:**

In July 2016, a 9-year-old girl (24 kg, 120 cm) was admitted with a pancreatic tail insulinoma and underwent robot-assisted spleen-preserving laparoscopic distal pancreatectomy. The total procedure time was 155 min, and the blood loss was about 10 ml. The patient recovered without complications.

**Conclusions:**

This case supports that robot-assisted spleen-preserving laparoscopic distal pancreatectomy may be safe and feasible in pediatric insulinoma patients.

## Background

Insulinomas are functional neuroendocrine pancreatic tumors that are mainly treated via surgical resection. The traditional open surgeries have been largely replaced by minimally invasive surgeries, including laparoscopic surgery and robot-assisted laparoscopic surgery (RAS). The Da Vinci surgical robot (Intuitive Surgical, Mountain View, CA, USA) offers the advantages of a three-dimensional (3D) surgical field and high-resolution viewing, which allow it to overcome difficulties in complicated surgeries better than conventional laparoscopic surgery. RAS is increasingly being practiced in the treatment of pediatric urologic conditions and digestive disease [1]. However, the use of RAS in pancreatic surgeries in children has not been reported. Here, we report our initial experience of robot-assisted, spleen-preserving laparoscopic distal pancreatectomy in a 9-year-old girl with insulinoma.

## Case presentation

**Fig. 1:**
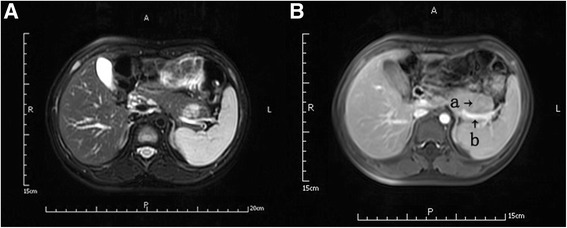
Preoperative abdominal dynamic contrast-enhanced MRI showing tumor at pancreas tail. **A** T2-weighted MR image showing loss of the normal shape of the pancreas tail and the clear margin of the tumor. **B** The nutrient vessel stems from the spleen vein. a nutrient vessel; b spleen vein

A 9-year-old Chinese girl was admitted to our unit due to episodic fainting attacks associated with sweatiness and decreased consciousness persisting for a year with aggravation for the previous 1 month. Hypoglycemia with a serum glucose level of 2–3 mmol/L was confirmed when the symptoms appeared and was resolved with intravenous glucose infusion. The fasting plasma blood-glucose level (FBG) was 1.84 mmol/L, insulin level was 8.2 mU/L, and insulin/glucose ratio was 4.46. An abdominal dynamic contrast-enhanced magnetic resonance imaging (MRI) scan revealed a tumor at the tail of the pancreas (Fig. 1A, B). There were no abnormal findings on ultrasonography of the thyroid gland and adrenal gland. Considering the case history, laboratory data, and imaging findings, the patient was diagnosed with insulinoma in the tail of the pancreas, and robot-assisted, spleen-preserving laparoscopic distal pancreatectomy was planned.

The patient was given a 5% glucose intravenous infusion constantly and slowly to prevent hypoglycemia. The robot was placed at the head of the patient. The assistant surgeon was positioned between the patient’s legs, and the surgical nurse stood at the right side of the patient. The patient’s blood glucose level was monitored and kept above 3.4 mmol/L throughout the whole procedure.

**Fig. 2:**
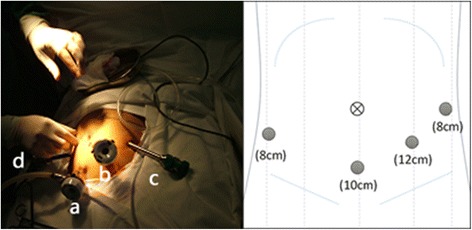
Placements of four trocars. a lens hole, b assistant hole, c arm 1, and d arm 2

The pneumoperitoneum pressure was 12 mmHg. A total of four trocars were used. The first trocar (10 cm) was inserted 5 cm below the umbilicus. The other three trocars were introduced under laparoscopic watch into the left anterior axillary line at the umbilical level (8 mm), the right anterior axillary line 2 cm below umbilical level (8 mm), and the left midclavicular line 3 cm below the umbilical level (12 mm; Fig. 2).

**Fig. 3:**
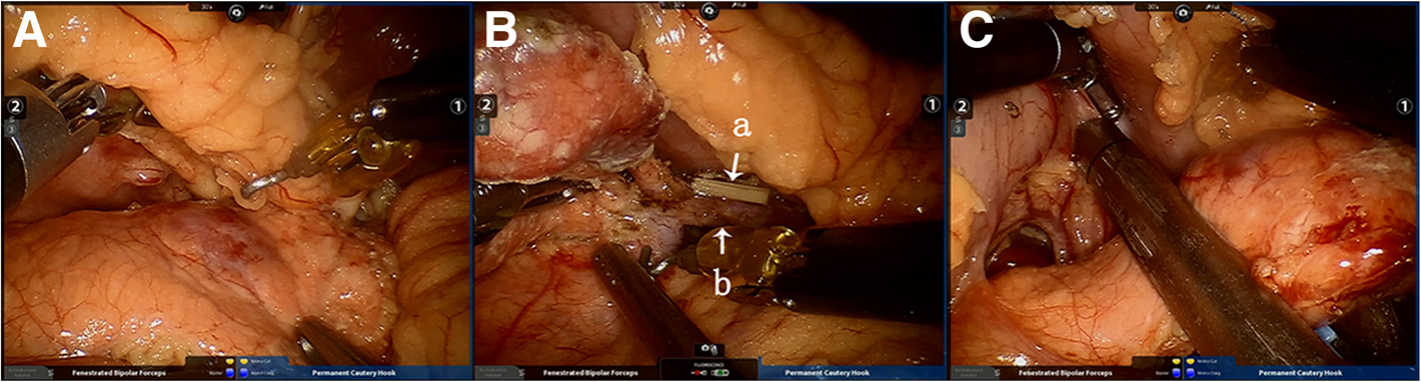
Operative images during robot-assisted spleen-preserving laparoscopic distal pancreatectomy. **A** The tumor was seen at the tail of the pancreas. **B** Clamping the nutrient vessel from the spleen vein. a nutrient vessel; b spleen vein. **C** EC60 (3.5 mm), the structure of the pancreas body and tail remained the same, but the body and tail of the pancreas lost their normal shape, and the tumor was a local bulge-like shape, with hyperemia on surface

The surgical procedure was performed as follows: After the gastrocolic ligament was dissected with an ultrasound knife, the posterior gastric wall was sutured to the round ligament of the liver with 4-0 prolene in order to expose the pancreas. Then, the gastrocolic ligament and splenocolic ligament were dissected to move the splenic flexure of the colon away from the surgical field. A hard tumor was seen at the tail of the pancreas, with a diameter of 2 cm, purplish color, and a clear margin (Fig. 3A). Laparoscopic ultrasonography showed main pancreatic duct involvement, and no other pancreatic lesion was found. The spleen vein was seen embedded with the pancreatic parenchyma; both the surgeon and assistant separated them carefully, patiently, and gently. Then, the pancreatic capsule was dissected along the lower edge of the pancreas with a hook knife, and the splenic vessels were separated and divided from the pancreatic cyst to avoid bleeding caused by adherence (Fig. 3B). After complete isolation of the tail of the pancreas from the spleen, the ECHELON endoscopic linear stapler (EC 60A, Johnson & Johnson, OH, USA) was used to transect 1 cm proximal to the tumor (Fig. 3C). The antecedent 4-0 prolene in the posterior gastric wall was cut, and a suction drain was placed through the lesser sac to the transected pancreas. Fast–frozen pathology indicated a neuroendocrine pancreatic tumor, or insulinoma, considering the clinical symptoms. The total operating time was 155 min, in which the pneumoperitoneum time was 120 min with an estimated blood loss of 10 ml.

**Fig. 4:**
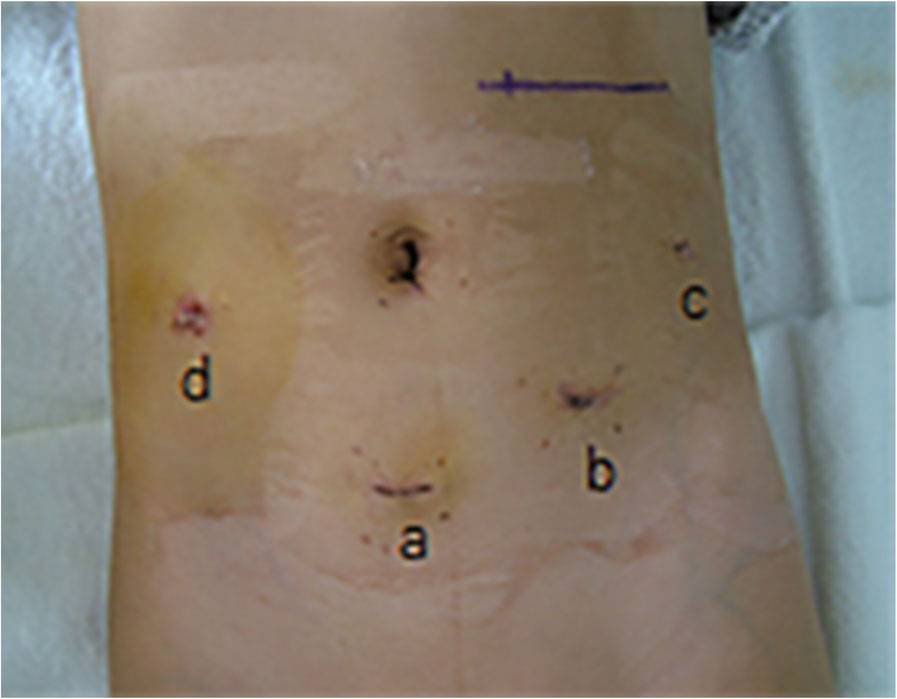
Wound condition on day 7

The urinary catheter was removed on day 1. The gastrointestinal decompression was successfully removed on day 3. The drain was clean and removed on day 7 (Fig. 4). The patient was given solid food on day 7 and discharged to home on day 8 after abdominal MRI showed no abnormalities. Her FBG was kept above 3.4 mmol/L pre-operatively, 12 mmol/L after removal of the tumor, and 9 mmol/L after the surgery.

The post-operative pathology results indicated a neuroendocrine tumor in the pancreas tail, 2 × 2 × 1.2 cm, mitosis count 3/10 HPF, IHC: Ki-67 (+10%), CgA (+), Syn (3+), SSTR2 (−), CK (+), CD56 (+), SSTR3 (+), SSTR1 (+), β-catenin (+), CD117 (-), and Grade 2, with local nerve involvement.

## Discussion and conclusions

Pancreatic insulinoma is an uncommon neuroendocrine tumor with an estimated incidence of 4 per 1,000,000 people worldwide [2]. Most insulinomas are benign and are seen only in children older than 4 years. The classic clinical symptoms are known as “whipple’s triad,” which often begin with psychiatric symptoms. In this case, the insulin/glucose ratio was 4.46 when the patient was experiencing hypoglycemia. Ultrasonography of the thyroid gland and adrenal gland cleared multiple endocrine neoplasia type 1 (MEN1).

Most pediatric insulinomas are curable by surgical removal of the lesion [3]. Most insulinomas are benign, and local resection is achievable. When the tumor is located in the pancreatic surface and encapsulated completely, local enucleation is feasible. Distal pancreatic resection is suitable for the pancreatic body and tail, especially when the tumor is large in size or near the main pancreatic duct. In addition, pancreatoduodenectomy is needed when a lesion is located in the head of the pancreas.

Distal pancreatectomy includes pancreatosplenectomy, spleen-preserving distal pancreatectomy, and vessels-sparing spleen-preserving distal pancreatectomy. The vessels-sparing spleen-preserving distal pancreatectomy can fully retain the physiological structure and function of the spleen to prevent postoperative thrombocytosis, thrombosis, pneumonia diplococcus infection, spleen infarction, spleen abscess, gastric varices, and other complications. It is applicable to benign, small, or well-differentiated tumors on the pancreas tail. However, the pancreas is adjacent to many important organs and vessels, and thus, the practice of this surgery is difficult and high risk.

The Da Vinci surgical robot can provide a 3D, clear, and wide surgical field, with a magnification of ×10–15. It can also complete surgical clamping, grasping, cutting, and suturing effectively and accurately with a great degree of simulation and jittering filtration, which shows an obvious advantage of limiting the surgical field during pediatric abdominal surgeries [4]. This technology has been applied in pediatric urological surgeries and in simple procedures for laparoscopic abdominal and thoracic surgeries, such as gastric folding, common bile duct cyst resection, biliary enteric anastomosis, esophageal resection, etc. However, the robot-assisted laparoscopic operation for pediatric pancreatic surgery has not been reported to the best of our knowledge.

With the accumulation of experience, robot-assisted laparoscopic proximal pancreatic resection, central pancreatic resection, and pancreatic tumor enucleation are feasible [5]. Compared with laparoscopic and open surgery, robot-assisted laparoscopic pancreatic surgeries are preferable due to the increase in the negative margin resection rate and retention rate of spleen and reduced intraoperative blood loss and hospital stays [6]. Preliminary research showed that the robot-assisted laparoscopic pancreatectomy was equally safe with laparoscopic surgery, and it is as safe and feasible for the benign and borderline tumors in the left pancreas [7, 8]. However, there were no experts to carry out a robot-assisted laparoscopic, spleen-preserving pancreatectomy for pediatric insulinoma.

For adult patients, trocars are mainly inserted in the upper abdomen with conventional 5-trocar placements as the abdominal wall area is larger. Children are shorter and the area of the abdominal wall is smaller. Thus, the trocars are placed below the umbilical level. For the present case, we placed the trocars below the umbilicus, and the surgical field was in the left upper quadrant. We arranged the auxiliary holes in the left lower abdomen, with arm 1 and arm 2 in left and right lower abdomen, respectively. The cold light source lens was placed in the middle abdomen pubic symphysis. All the procedures were completed through the four holes without unwanted contact of the equipment.

We believe that the precondition for successful robot-assisted laparoscopic, spleen-preserving distal pancreatectomy for pediatric insulinoma is an accurate surgical approach, which minimizes the interference of tissue around the tumor. In this case, the tumor was located in the pancreatic body tail, which was about 3.0 cm in diameter, and there was a certain border to the pancreatic tissue. The body and tail of the pancreas lost their normal shape, and the tumor was a localized bulge-like shape and with hyperemia on surface. Thus, local enucleation was not suitable, and the distal pancreatectomy was appropriate.

We began using the Da Vinci Robot 5 years ago in 2011. To date, we have accumulated a large number of surgical experiences, more than 500 cases, of which more than 300 cases require pancreatic surgery. The success of this initial experience revealed that the robot-assisted laparoscopic surgeries have the advantages of 3D visual field and flexible operation over conventional laparoscopic surgeries, which lead to a higher chance of surgical success. This case showed that robot-assisted spleen-preserving distal pancreatectomy is safe and feasible for children with insulinoma.
